# Development of a rare disease algorithm to identify persons at risk of Gaucher disease using electronic health records in the United States

**DOI:** 10.1186/s13023-023-02868-2

**Published:** 2023-09-09

**Authors:** Amanda Wilson, Alexandra Chiorean, Mario Aguiar, Davorka Sekulic, Patrick Pavlick, Neha Shah, Lisa Sniderman King, Marie Génin, Mélissa Rollot, Margot Blanchon, Simon Gosset, Martin Montmerle, Cliona Molony, Alexandra Dumitriu

**Affiliations:** 1 Health Economics and Value Assessment, Sanofi, Cambridge, MA USA; 2 Quinten Health, Paris, France; 3 Global Medical Affairs, RD Hematology, Sanofi, Cambridge, MA USA; 4 US Rare Medical, Sanofi, Cambridge, MA USA; 5 Medical Diagnostics, Sanofi, Cambridge, MA USA; 6 Digital Data Science, Sanofi, Cambridge, MA USA; 7 Global Medical Affairs, Medical Evidence Generation, Sanofi, Cambridge, MA USA

**Keywords:** Electronic health records, Gaucher disease, Machine learning, Patient identification, Real-world evidence

## Abstract

**Background:**

Early diagnosis of Gaucher disease (GD) allows for disease-specific treatment before significant symptoms arise, preventing/delaying onset of complications. Yet, many endure years-long diagnostic odysseys. We report the development of a machine learning algorithm to identify patients with GD from electronic health records.

**Methods:**

We utilized Optum’s de-identified Integrated Claims-Clinical dataset (2007–2019) for feature engineering and algorithm training/testing, based on clinical characteristics of GD. Two algorithms were selected: one based on age of feature occurrence (age-based), and one based on occurrence of features (prevalence-based). Performance was compared with an adaptation of the available clinical diagnostic algorithm for identifying patients with diagnosed GD. Undiagnosed patients highly-ranked by the algorithms were compared with diagnosed GD patients.

**Results:**

Splenomegaly was the most important predictor for diagnosed GD with both algorithms, followed by geographical location (northeast USA), thrombocytopenia, osteonecrosis, bone density disorders, and bone pain. Overall, 1204 and 2862 patients, respectively, would need to be assessed with the age- and prevalence-based algorithms, compared with 20,743 with the clinical diagnostic algorithm, to identify 28 patients with diagnosed GD in the integrated dataset. Undiagnosed patients highly-ranked by the algorithms had similar clinical manifestations as diagnosed GD patients.

**Conclusions:**

The age-based algorithm identified younger patients, while the prevalence-based identified patients with advanced clinical manifestations. Their combined use better captures GD heterogeneity. The two algorithms were about 10–20-fold more efficient at identifying GD patients than the clinical diagnostic algorithm. Application of these algorithms could shorten diagnostic delay by identifying undiagnosed GD patients.

**Supplementary Information:**

The online version contains supplementary material available at 10.1186/s13023-023-02868-2.

## Background

Rare inherited lysosomal storage disorders (LSDs) are a heterogeneous group of about 70 monogenic disorders characterized by defects in lysosomal function [1, 2]. These disorders affect multiple organs and systems, leading to a broad range of progressive clinical manifestations that are often highly debilitating and shorten lifespans. However, manifestations are highly variable depending on the location and extent of lysosomal storage, both within and between disorders [3]. Those with LSDs often need lifelong care, though early diagnosis can crucially allow treatment of some LSDs before significant symptoms arise, preventing or delaying onset of complications [3, 4].

Gaucher disease (GD) is an LSD caused by deficiency in the lysosomal enzyme glucocerebrosidase [5, 6]. Glucocerebrosidase deficiency results in progressive glucosylceramide accumulation predominantly in the spleen, liver, and bone marrow, although other organs may also be affected, leading to a wide range of symptoms, including hepatosplenomegaly, anemia, thrombocytopenia, bone lesions/symptoms, and neurological impairment [5]. GD is categorized into three phenotypes: type 1 or non-neuronopathic; type 2 or acute neuronopathic; and type 3 or chronic neuronopathic, which differ by the presence or absence, extent, and rate of progression of neurodegeneration [7, 8]. GD affects about 1 in 40,000–100,000 live births in the general population but occurs at a higher frequency (1 in 500–1000) among those of Ashkenazi Jewish descent [9]. The National Organization for Rare Disorders estimates that there are about 6000 individuals with GD in the USA [10], but estimates as high as 20,000 individuals have also been suggested [11]. The incidences of GD, and LSDs in general, are likely underestimated because of limited disease awareness, leading to many patients remaining undiagnosed, or misdiagnosed and receiving inappropriate investigations and/or interventions [12–14].

GD is a heterogeneous disease, and each patient is unique regarding age of onset and range of symptoms, rate of disease progression, and comorbidities. Early GD-specific features tend to reflect hematological aspects of the disorder, with many patients initially referred to hematologists [15]. Often, among those presenting with the classic GD symptoms (cytopenia, hepatosplenomegaly, bone pain) to hematologists/oncologists, initial misdiagnoses include leukemia, lymphoma or multiple myeloma [14]. Thus, patients often have long diagnostic journeys over many years involving various specialists and tests [15], worsening outcomes. Early recognition of symptoms and treatment may reduce the incidence of severe and irreversible long-term sequelae of GD [16]. Widespread genetic screening of newborns for rare diseases will help avoid the diagnostic odyssey and improve outcomes for those identified, but is not yet widely available for many rare diseases [17]. Furthermore, there is a need to identify undiagnosed adult patients, including those with less severe forms of GD, that could benefit from treatment later in life.

A clinical diagnostic algorithm is available for GD [18]. Although effective, its usefulness in practice relies on physician awareness of the disorder, knowledge of the clinical diagnostic algorithm, as well as verification of numerous clinical characteristics, including differential diagnoses. The development of algorithms using machine learning to identify patients who are highly suspected of having rare disorders can alleviate some of the limitations by reducing the a priori deduction required to apply the clinical diagnostic algorithms. Indeed, the ability of machine learning algorithms to incorporate a wide array of clinical characteristics can enhance the diagnostic abilities of clinicians to identify individuals with complex presentations of rare diseases.

Here, we report on the development of two algorithms using machine-learning techniques to identify highly suspected patients with GD in Optum’s de-identified Integrated Claims-Clinical dataset.

## Methods

### Data source

We utilized Electronic Health Records (EHRs) contained within Optum’s de-identified Integrated Claims-Clinical dataset (2007–2019) for exploratory analysis, feature engineering, feature selection, and algorithm training, tuning, selection, and testing. Optum^®^ Integrated database aggregates de-identified EHR data from providers across the continuum of care. It is derived from dozens of US healthcare provider organizations, that include > 700 hospitals and > 7000 clinics, treating > 102 million patients. This study did not require oversight by an independent institutional review board since only de-identified patient data were used.

### Study populations

#### GD cohort

The GD cohort was defined as patients with at least two instances of GD diagnoses (ICD-10 code E75.22) or at least one record of a GD-specific treatment (Table S1) in the integrated database. The index date was set as the first day of the initial diagnosis or receipt of treatment. The look-back period for patients with GD was defined as the duration of coverage prior to the index date.

Patient records were excluded if they had incoherent diagnosis or treatment timelines in relation to the index date (e.g. reported first date of activity after index date, reported last date of activity before index date, or death before index date). Patient records with other LSDs (such as Fabry, Pompe, Niemann-Pick, Tay-Sachs or mucopolysaccharidosis) were also excluded due to the potential for coding errors in patients with different LSDs.

#### Control cohort

The control cohort was selected from the remaining records of patients who did not meet the GD inclusion criteria, subject to the same exclusion criteria, except for the presence of another LSD. The index date for the controls was set at the median look-back period for the GD cohort.

### Feature identification and definitions

We identified four categories of features to use in the model: (1) clinical characteristics of GD based on a review of the scientific literature, including information from the Genetic and Rare Diseases Information Center (GARD) [19]—80 clinical characteristics of GD were identified and grouped into organ classes (Additional file 1: Fig. S1); (2) three demographic features, comprising race (African American, Asian, Caucasian, other/unknown), US region (midwest, south, west, northeast, other/unknown) and gender (male, female); (3) information on 16 interactions with healthcare system “providers” (specialists visited [primary care, medical genetics, neurology, hematology, oncology, rheumatology, ophthalmology, internal medicine, general practice, orthopedic surgery, hepatology, gastroenterology, pediatrics, pain medicine, radiology, family medicine]) by “visits” (type of patient visit [emergency, inpatient, outpatient]) and “encounter” (all patient interaction types [such as home visits, imaging]); (4) eight data-driven features derived to include features prevalent in the GD cohort that were not captured by the scientific literature review.

To assess data-driven features, Cramer’s V test was used to identify features that were more, or less, frequently associated with the GD cohort than controls. A separate control cohort was identified for this exercise (control patients selected for algorithm training were not considered for this step), using an exact matching (1:100 GD/control ratio) without replacement based on years of coverage. This created a similar control population to the GD cohort. Expert clinical opinion was used to trim features that are likely false associations and/or irrelevant to the clinical picture. Features selected were those with Cramer’s V coefficients > 0.1 threshold and *p* values < 0.05 using a Chi-square test.

Once the features were determined, we defined each feature using information from the de-identified EHR. Four data sources within the EHR were used to define the features: (1) diagnosis codes, specifically International Classification of Diseases 9th and 10th Revisions (ICD-9 and ICD-10) diagnosis codes; (2) procedures, specifically ICD-9 and ICD-10 procedure codes; (3) laboratory measurements; (4) pre-extracted Signs/Disease/Symptoms (SDS) terms using natural language processing from providers’ notes. Additional file 1: Fig. S2 illustrates how information from these data sources was combined to define the features.

Two ways of encoding features were defined: age at first occurrence (or if not presented, censured beyond a reasonable human lifetime at 200 years) (age-based) or binary presence/absence of the feature (prevalence-based). Features of healthcare interactions were also encoded by frequency of encounters. Treatments were only derived with binary flags (presence/absence).

### Algorithm selection, training and assessment

To ensure appropriate patients would be used to train the algorithm, hierarchical agglomerative clustering was used to identify non-representative patients with GD with a paucity of information for removal from algorithm training [20, 21]. The GD cohort identified for algorithm training was restricted to those with at least 1 year coverage. Events across the study period (i.e. before and after the index date) were considered for training to account for disease evolution after diagnosis, with exceptions for events expected to be biased after diagnosis (e.g. visits).

Light Gradient Boosting Machine (LightGBM) [22] was used to develop the algorithms to predict the likelihood of GD using all the features described above. The algorithms were trained on a 1:10 GD to control training ratio (training cohort). Random sampling was performed to select 10 patients without GD among a cohort composed of 500 patients without GD for 1 patient with GD.

The trained algorithms were evaluated on a population containing both patients with GD and controls in a 1:10,000 GD to control ratio (test cohort); 100 patients with GD and 1 million controls independent from the training cohort were randomly picked based on the criteria previously defined. The trained algorithms were applied on the test dataset with no censoring of any event during the observation period, to assess how the algorithm would perform in conditions close to the real-life application (i.e. no information censored).

Algorithm performance was evaluated using the area under the precision and recall curve (AUPRC), a standard approach for imbalanced dataset classification [23]. Hyper-parameter tuning and optimization was performed on the AUPRC through a 10-folds cross validation, with a ratio of 1 patient with GD to 10 controls. Bootstrapping on controls was performed to limit sample bias during random selection. The cross-validation was performed 10 times, by selecting each time 10 controls for 1 patient with GD from the training dataset initially composed of 500 controls for 1 patient with GD. Controls were unique (no replacement) in each bootstrap, but could be selected in several bootstraps (selection with replacement between bootstraps). The distribution of the 10 best AUPRC (one for each bootstrap) was analyzed to ensure the robustness of the algorithm. The final algorithm was a randomly picked bootstrap among the 10 choices (i.e. a training dataset at 1 patient with GD to 10 control ratio and its associated best hyper-parameters determined with cross-validation).

The Shapley additive explanations (SHAP) method was utilized to understand the role each feature played in the algorithm predictions. The SHAP method assigns each feature an importance value for its contribution to a particular prediction probability [24], i.e. GD in our study. The larger the SHAP value, the higher the feature’s importance in prediction of the outcome. The prediction probability value of each algorithm for each patient and the sum of the algorithm values were used to rank patients as the most highly suspected of having GD. In order to assess the results, we chose a ≥ 0.95 prediction probability threshold to define the “highly suspected population”. In addition, we adapted the filter criteria from an existing clinical decision tool for the identification of patients with GD [18], to the integrated database using diagnostic/procedure codes and other identifiers (Additional file 1: Fig. S3) as an alternate method of identifying patients highly suspected of having GD.

The patient groups of interest (including the “highly suspected population”) were characterized with descriptive statistics by age distribution, prevalence of clinical characteristic or visits, age at first occurrence of clinical characteristics or visits, prevalence of treatments, and percentage of patients who received GD treatment or who had a differential diagnosis. The performance of the algorithms was compared with the clinical diagnostic algorithm by determining the number of patients needing diagnostic testing to find a given number of patients with GD [18]. The demographic and clinical characteristics of the GD “highly suspected population” identified by the age- and prevalence-based algorithms, as well as those (1) identified using the clinical diagnostic algorithm, and (2) the entire diagnosed GD cohort were described.

## Results

### Gaucher disease and control population

#### Patients

The diagnosed GD cohort comprised 829 patients (207 with ≥ 2 GD diagnoses, 248 with ≥ 1 GD treatment, and 374 with ≥ 2 GD diagnoses and ≥ 1 GD treatment). Of these, 14 were excluded: 2 died before the index date; 3 had missing age or gender information; and 9 were also diagnosed with other LSDs, indicating potential misdiagnosis. The final diagnosed GD cohort comprised 815 patients; of these, 100 were randomly selected and set aside for the test cohort. Of the remaining 715 that could be used to train the algorithm, 59 were not included because they had < 1-year coverage; thus, 656 were used for algorithm training. Therefore, a total of 756 patients with diagnosed GD were included in the training and testing of the algorithms. These were matched with 328,000 controls (500 controls for 1 patient with GD) to form the training cohort, and 1,000,000 controls (10,000 controls for 1 patient with GD) to form the test cohort, respectively, for a total of 1,328,000 controls. A summary of the demographics of the diagnosed GD and control cohorts utilized in our study is presented in Table 1.

**Table 1:** Demographics of the diagnosed GD and control cohorts

	Diagnosed with GD N = 756	Controls N = 1,328,000	*p* value^#^
**Age at index (years)**			< 0.001
Mean (SD)	44 (22)	40 (24)	
Min	0	0	
Median (Q1–Q3)	45 (27–61)	39 (21–58)	
Max	87	89	
**Age in class**			< 0.001
0–9	58 (7.7%)	175,841 (13.2%)	
10–19	57 (7.5%)	133,630 (10.1%)	
20–29	93 (12.3%)	181,418 (13.7%)	
30–39	107 (14.2%)	174,281 (13.1%)	
40–49	114 (15.1%)	167,545 (12.6%)	
50–59	121 (16.0%)	188,475 (14.2%)	
60–69	110 (14.6%)	148,014 (11.1%)	
70–79	72 (9.5%)	102,493 (7.7%)	
80–89	24 (3.2%)	56,303 (4.2%)	
**Race**			< 0.001
African American	29 (3.8%)	128,439 (9.7%)	
Asian	2 (0.3%)	28,957 (2.2%)	
Caucasian	624 (82.5%)	830,948 (62.6%)	
Other/unknown	101 (13.4%)	339,656 (25.6%)	
**Ethnicity**			< 0.001
Hispanic	37 (4.9%)	84,613 (6.4%)	
Not Hispanic	621 (82.1%)	877,906 (66.1%)	
Unknown	98 (13.0%)	365,481 (27.5%)	
**Region**			< 0.001
Midwest	193 (25.5%)	565,677 (42.6%)	
Northeast	345 (45.6%)	189,864 (14.3%)	
South	133 (17.6%)	352,187 (26.5%)	
West	61 (8.1%)	137,904 (10.4%)	
Other/unknown	24 (3.2%)	82,368 (6.2%)	
**Death**			
0	716 (94.7%)	1,258,157 (94.7%)	
1	40 (5.3%)	69,843 (5.3%)	
**Coverage (years)**			< 0.001
Mean (SD)	7 (3)	5 (4)	
Min	0.17	0	
Median (Q1–Q3)	7 (4–10)	4 (0–8)	
Max	12.75	12.82	
**Look-back period (years)**			< 0.001
Mean (SD)	3 (3)	2 (2)	
Min	0	0	
Median (Q1–Q3)	2 (1–5)	1 (0–2)	
Max	12.36	7.19	
**Look-back period**			
At least 6 months	573 (75.8%)	727,800 (54.8%)	
At least 12 months	513 (67.9%)	638,042 (48.0%)	
At least 18 months	452 (59.8%)	638,042 (48.0%)	
At least 24 months	404 (53.4%)	481,827 (36.3%)	
At least 36 months	325 (43.0%)	249,674 (18.8%)	
**Number of distinct symptoms**			< 0.001
Mean (SD)	5 (4)	2 (2)	
Min	0	0	
Median (Q1–Q3)	4 (2–7)	1 (0–2)	
Max	19	25	
**GD treatment**			
Untreated	0%		
Treated	0%		
**Rare disease history**			< 0.001
0	756 (100.0%)	1,327,978 (100.0%)	
1	0 (0%)	22 (0%)	

^#^Chi-square and *T* test for categorical and continuous variables, respectively.

#### Clinical characteristics and visits to specialists

The clinical characteristics of the diagnosed GD and control cohorts are presented in Table 2; the diagnosed GD cohort had a higher prevalence of most clinical characteristics including anemias, thrombocytopenia, bone density disorders, osteoarthritis, dysphagia, abdominal pain, fever, splenomegaly, pulmonary fibrosis, and respiratory failure (all *p* < 0.001, Chi-square and *T* test). In addition, the proportion who had visited the various specialists was also consistently higher in the diagnosed GD cohort (Table 3). The most significant specialists visited by diagnosed GD patients by visit or by age were oncologists (both *p* < 0.001, Chi-square and *T* test) or ophthalmologists (*p* < 0.001, Chi-square; *p* < 0.01, *T* test).

**Table 2:** Clinical characteristics of the diagnosed GD and control cohorts

Features	Diagnosed with GD			Controls			Chi-square	*T* test
	N = 756			N = 1,328,000				
	N	%	Age at 1st event; years, mean (SD)	N	%	Age at 1st event; years, mean (SD)		
**Anemia**								
Anemias	614	81.22	43 (21)	567,739	42.75	44 (22)	***	***
**Blood disorder**								
Coagulation defects	31	4.1	49 (25)	6922	0.52	56 (20)	***	
Pancytopenia	32	4.23	44 (25)	3794	0.29	62 (18)	***	
Thrombocytopenia	300	45.73	43 (22)	26,644	8.12	57 (21)	***	***
**Bone disorder**								
Arthralgia	19	2.51	48 (18)	12,932	0.97	56 (18)	***	
Arthrogryposis	9	1.19	68 (11)	9882	0.74	53 (19)		
Avascular necrosis	31	4.1	48 (18)	926	0.07	55 (18)	***	
Bone density disorders	229	30.29	54 (17)	49,065	3.69	67 (13)	***	***
Bone pain	65	8.6	43 (18)	2958	0.22	50 (21)	***	
Chondropathies	10	1.32	52 (24)	8913	0.67	48 (20)	*	
Delayed skeletal maturation	11	1.46	48 (22)	3908	0.29	56 (20)	***	
Erlenmeyer flask deformity	0	0		827	0.06	45 (20)		
Joint dislocation	2	0.26	58 (5)	3013	0.23	43 (21)		
Kyphosis	15	1.98	43 (29)	3709	0.28	62 (22)	***	*
Osteoarthritis	150	19.84	60 (15)	112,928	8.5	63 (14)	***	***
Osteolysis	5	0.66	66 (7)	203	0.02	58 (19)	***	
Osteonecrosis	50	6.61	47 (18)	1464	0.11	56 (17)	***	
Osteopenia	77	10.19	48 (19)	11,140	0.84	66 (12)	***	
Osteoporosis	58	7.67	58 (15)	13,119	0.99	68 (13)	***	
Pathological fracture	11	1.46	53 (18)	2283	0.17	65 (19)	***	
Spine deformation	0	0		136	0.04	40 (29)		
**Cerebral/nervous system disorder**								
Ataxia	10	1.32	51 (29)	4534	0.34	62 (19)	***	
Bradykinesia	4	0.53	62 (11)	359	0.03	70 (13)	***	
Cranial nerve disorders	1	0.13	84	267	0.02	55 (20)		
Developmental regression	51	6.75	36 (29)	34,475	2.6	39 (31)	***	**
Dysphagia	57	7.54	50 (27)	34,106	2.57	58 (21)	***	***
Extrapyramidal disorder	5	0.66	53 (15)	1660	0.13	52 (21)	***	
Gaze palsy	1	0.13	46	77	0.01	49 (27)	*	
Hearing impairment	66	8.73	55 (23)	57,555	4.33	53 (25)	***	**
Hemiplegia/hemiparesis	7	0.93	55 (22)	6215	0.47	63 (19)		*
Hydrocephalus	6	0.79	60 (29)	2078	0.16	50 (28)	***	
Laryngeal spasm	3	0.4	29 (41)	468	0.04	40 (27)	***	
Muscle hypotonia	7	0.93	3 (2)	587	0.04	9 (17)	***	
Myoclonic seizure	1	0.13	17	144	0.01	30 (19)		
Nerve root compression	0	0		419	0.03	56 (17)		
Oculomotor apraxia	4	0.53	28 (36)	286	0.02	39 (29)	***	
Opticokinetic nystagmus	5	0.66	26 (32)	1126	0.08	40 (26)	***	
Paralytic strabismus	1	0.13	15	676	0.05	28 (26)		
Parkinson	22	2.91	64 (11)	3434	0.26	73 (11)	***	
Tonic clonic seizure	1	0.15	9	159	0.05	42 (23)		
**Development disorders**								
Delayed puberty	1	0.13	13	292	0.02	16 (9)		
Growth retardation	6	0.79	15 (13)	219	0.02	11 (14)	***	**
Short stature	7	0.93	11 (4)	1908	0.14	11 (11)	***	
**Eye disorder**								
Corneal disorders	7	0.93	49 (32)	2396	0.18	58 (20)	***	
Non diabetic retinopathy	3	0.4	61 (26)	2232	0.17	50 (28)		
**General signs**								
Abdominal pain	217	28.7	47 (22)	227,908	17.16	42 (22)	***	***
Elevated CRP	5	0.66	52 (16)	2204	0.17	50 (21)	**	
Fatigue	222	29.37	47 (19)	111,605	8.4	51 (21)	***	*
Fever	143	18.92	41 (23)	171,634	12.92	34 (25)	***	***
Gingival bleeding	2	0.26	51 (32)	230	0.02	42 (28)	***	
Muscle atrophy	4	0.53	77 (12)	120	0.01	61 (16)	***	
Tremor	27	4.12	53 (22)	4153	1.27	56 (20)	***	
Vitamin D deficiency	116	17.68	50 (19)	20,321	6.2	54 (18)	***	*
**Heart disorder**								
Valve calcification	9	1.37	72 (14)	1740	0.53	69 (14)	**	
**Hepatic disorders**								
Cirrhosis	14	1.85	46 (16)	5889	0.44	60 (13)	***	
Hepatic fibrosis	6	0.79	48 (29)	514	0.04	55 (15)	***	
Hepatitis	19	2.51	49 (23)	4761	0.36	54 (17)	***	
Portal hypertension	13	1.72	38 (22)	2734	0.21	59 (13)	***	
**Immunology**								
Polyclonal gammopathy	4	0.53	60 (19)	308	0.02	52 (22)	***	
**Kidney disorder**								
Acute kidney disease	31	4.1	66 (14)	4214	0.32	56 (20)	***	
Hematuria	74	9.79	53 (21)	61,349	4.62	52 (21)	***	*
Proteinuria	34	4.5	53 (22)	18,060	1.36	53 (21)	***	
**Malignancy**								
Liver neoplasm	3	0.4	55 (26)	1452	0.11	63 (14)		
Malignant melanoma	5	0.66	63 (7)	3092	0.23	62 (15)	*	
Multiple myeloma	7	0.93	61 (10)	1213	0.09	67 (13)	***	
Non Hodgkin Lymphoma	8	1.06	61 (27)	1738	0.13	65 (16)	***	
Other malignant neoplasms	2	0.26	38 (46)	150	0.01	56 (21)	***	
Pancreatic cancer	2	0.26	55 (15)	1381	0.1	67 (13)		
Uncertain neoplasms	41	6.25	58 (16)	1259	0.38	60 (19)	***	
**Organomegaly**								
Hepatomegaly	134	17.72	35 (22)	6230	0.47	52 (18)	***	**
Splenomegaly	238	36.28	39 (21)	2036	0.62	52 (19)	***	***
Ventriculomegaly	2	0.3	74 (0)	15	0	58 (26)	***	
**Perinatal disorders**								
Hydrops fetalis	1	0.13	2	34	0	21 (15)	***	
Ichthyosis	0	0		221	0.02	44 (27)		
**Psychiatric disorders**								
Dementia non-senile	19	2.51	69 (16)	17,888	1.35	78 (10)	**	***
Depression	159	21.03	50 (20)	165,339	12.45	46 (20)	***	**
**Respiratory disorder**								
Interstitial pulmonary abnormality	7	0.93	44 (36)	2600	0.2	67 (16)	***	**
Pulmonary fibrosis	13	1.72	63 (20)	6865	0.52	67 (15)	***	***
Pulmonary hypertension	33	4.37	58 (20)	11,173	0.84	69 (17)	***	
Respiratory failure	35	4.63	57 (29)	27,676	2.08	63 (20)	***	***

*p value < 0.05

**p value < 0.01

***p value < 0.001

**Table 3:** Visits to specialist by the diagnosed GD and control cohorts

Features	Diagnosed with GD N = 756				Controls N = 1,328,000				Chi-square	*T* test (no. visit)	*T* test (age)
	N	%	No. of visits (mean)	Age at 1st event; years, mean (SD)	N	%	No. of visits (mean)	Age at 1st event; years, mean (SD)			
Gastroenterology	70	9.26	1.4	50 (21)	56,098	4.22	1.69	53 (18)	***		
General Practice	10	1.32	19.11	52 (21)	13,816	1.04	2.71	41 (23)			
Hematology	10	1.32	6.05	56 (15)	1455	0.11	2.7	54 (17)	***		
Hepatology	4	0.53	1.18	42 (15)	643	0.05	2.55	51 (17)	***		
Internal Medicine	190	25.13	2.03	51 (18)	176,762	13.31	3.46	49 (20)	***		
Neurology	186	24.6	3.55	42 (21)	64,235	4.84	2.71	45 (22)	***		
Oncology	131	17.33	6.42	43 (23)	24,644	1.86	4.18	53 (21)	***	***	***
Ophthalmology	47	6.22	1.09	10 (21)	40,409	3.04	1.87	49 (23)	***	**	**
Orthopedic Surgery	107	14.15	1.81	49 (21)	62,527	4.71	2.69	48 (21)	***		
Pain Medicine	10	1.32	0.42	47 (23)	5822	0.44	1.81	53 (16)	***		
Pediatrics	68	8.99	4.93	12 (16)	90,238	6.8	5.97	10 (15)	*		
Radiology	119	15.74	1.54	48 (23)	91,892	6.92	1.58	46 (21)	***		
Rheumatology	23	3.04	0.66	60 (18)	13,402	1.01	2.18	54 (17)	***		

*p value < 0.05

**p value < 0.01

***p value < 0.001

### Training and test cohorts

The distributions of the characteristics among the training and test cohorts were assessed to verify whether bias was introduced between the two cohorts due to the different cohort sizes, which would cause the algorithm to behave differently during testing compared to training.

The diagnosed GD cohort assigned to the training (656 patients) and the test datasets (100 patients) had on average the same age (44 years), the same coverage (7 years) and a similar number of symptoms (5 symptoms). However, by chance, those in the training dataset appeared to have lower prevalence of visits than those in the test dataset. For example, patients with GD from the training dataset had fewer visits to an internal medicine specialist (25% vs. 58%, 2 visits vs. 48 visits on average), a neurologist (25% vs. 47%, with 3.8 visits on average vs. 47 visits on average) and a radiologist (16% vs. 46%).

The control training (328,000 patients) dataset overall appeared to have more severe symptoms than those in the control test (1,000,000 patients) dataset but had fewer visits due to post-index date censorship. Those in the training dataset had a higher coverage period (average 7 ± 3 vs. 6 ± 3 years), were more symptomatic (average number of symptoms 5 [± 4] vs. 2 [± 2]), and had a higher prevalence of anemias (54% vs. 39%) and abdominal pain (22% vs. 15%) than the test dataset.

### Algorithm selection

Two final algorithms to predict the likelihood of GD were retained, one where feature encoding was defined by age at first occurrence (age-based algorithm) and the other where feature encoding was binary presence/absence (prevalence-based algorithm). The AUPRC was 0.66 for both algorithms. AUPRC can range from 0 to 1, where the baseline (equivalent to a random classifier) is equal to the fraction of positives [25]. In our case, the baseline AUPRC would be 0.1 given the 1:10 GD to control ratio in the training set. Therefore, the performance of both algorithms is 6.6 times better in predicting the likelihood of GD than a baseline classifier.

### Patients identified with suspected GD by the algorithms and the currently available clinical diagnostic algorithm

The demographics and clinical characteristics of the “highly suspected population” with GD identified by the two algorithms, as well as their visits to the specialist, are summarized in Additional file 1: Tables S2–S4, along with those identified using the currently available clinical diagnostic algorithm as having suspected GD, and the entire diagnosed GD cohort.

In general, the two algorithms identify different types of patients. Those identified with the age-based algorithm were younger than those identified with the prevalence-based algorithm (mean age, 36 vs. 52 years) (Additional file 1: Table S2), and those identified by the latter tended to have more clinical features present but with first occurrence appearing at a later age (Additional file 1: Table S3). Although those identified with the prevalence-based algorithm also generally had higher prevalence of visits to specialists than those identified by the age-based algorithm, they tended to have fewer mean visits (Additional file 1: Table S4).

In comparison to both the age- and prevalence-based algorithms, those identified using the clinical diagnostic algorithm (Additional file 1: Fig. S3) were older patients (mean 61 years), tended to have more symptoms, and the mean age of symptom onset was generally later than with the two algorithms (Additional file 1: Table S2 and S3). However, they had a lower prevalence of organomegaly (Additional file 1: Table S3) and their first visit to the specialist was at an older age (Additional file 1: Table S4).

### Feature importance

**Fig. 1:**
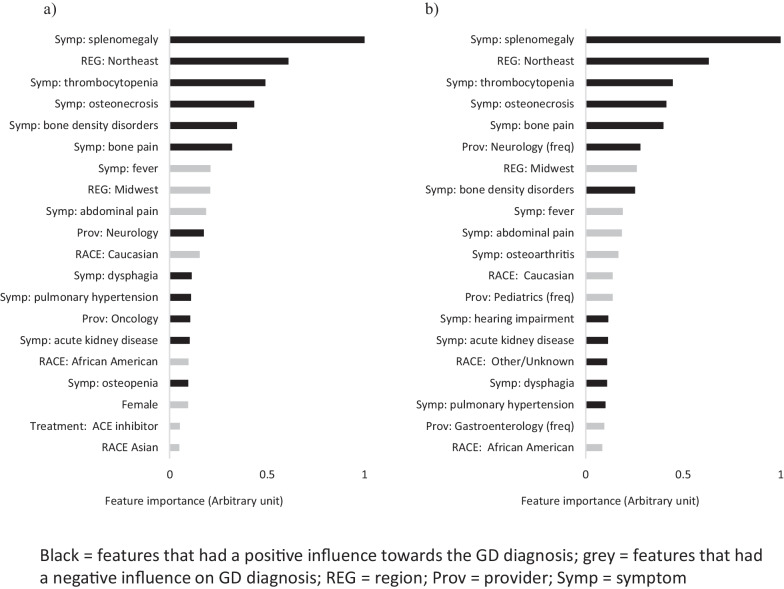
Comparison of top drivers of GD prediction: **a** age and **b** prevalence algorithms. The feature importance presented is the average influence from all patients identified; splenomegaly was the most important feature and was set at an arbitrary value [AV] of 1, from which all the other feature relative importance values were calculated

The top GD prediction drivers in the age- and prevalence-based algorithms are summarized in Fig. 1. The top four most important features and their relative importance ranking were the same across both algorithms; these were splenomegaly, the patient being located in the northeast region, thrombocytopenia and osteonecrosis; all increased the probability of predicting GD. Bone density, bone pain and frequency of visits to the neurologist were among the top ten most important features, which also increased the probability of predicting GD, though their relative importance ranking differed between the two algorithms. Fever, the patient’s location as the midwest region and abdominal pain were also among the top ten most important features, but these decreased the probability of predicting GD.

### Assessing algorithm performance—comparison with real world application of the available clinical diagnostic algorithm

**Fig. 2:**
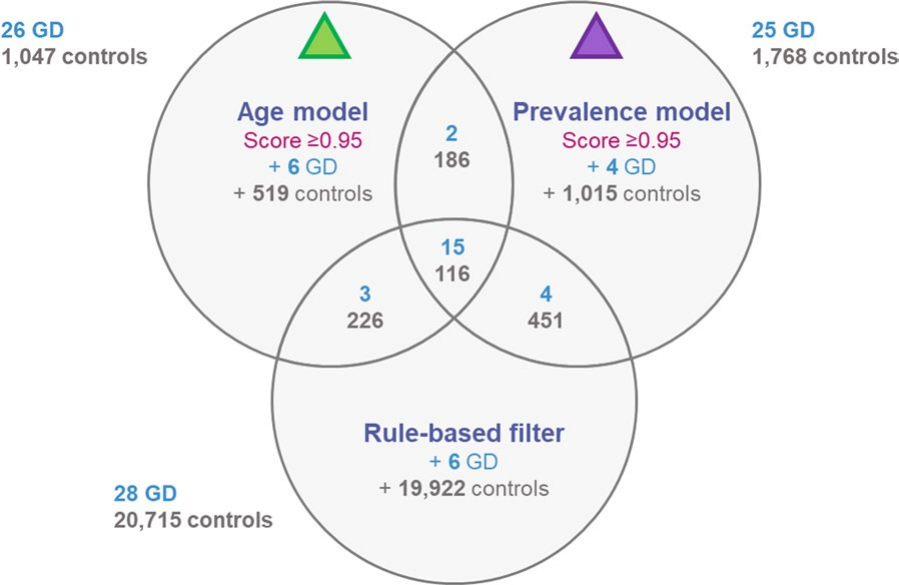
Numbers of patients with diagnosed GD identified by the algorithms, among those identified as a “highly suspected population” with GD (threshold > 0.95) by the age- and prevalence-based algorithms, and those identified using the clinical diagnostic algorithm as having suspected GD. The numbers shown outside the circles are those identified by the respective models who had a GD diagnosis already (in blue) and as the “highly suspected population” with GD (in grey)

The number of patients with diagnosed GD identified by both algorithms (among the “highly suspected population”) in relation to each other, and among those identified using the clinical diagnostic algorithm is summarized in Fig. 2. Overall, 1204 and 2862 patients would be required to be assessed with the age- and prevalence-based algorithms, respectively, versus 20,743 with the clinical diagnostic algorithm, to identify 28 patients with diagnosed GD.

## Discussion

We used real-world data to develop two algorithms to identify patients with a high likelihood of GD, who would be appropriate candidates for confirmatory diagnostic testing. As expected, the top drivers for prediction of GD in both the age- and prevalence-based algorithms included splenomegaly and thrombocytopenia, which are among the ‘hallmarks’ of GD. Although splenomegaly and thrombocytopenia could also be attributed to infectious diseases such as HIV, we preferred not to include differential diagnoses in the algorithm training. Indeed, since several signs and symptoms are common between GD and HIV (e.g. splenomegaly and thrombocytopenia), HIV could be mistaken by the algorithm as being associated with GD. Therefore, HIV was excluded from training, but kept as descriptive information of the cohorts. If the algorithm was applied, this could be done as a post-filtering step. Of note, the northeast region was also a top predictor of GD with both algorithms. The northeast region has the highest Jewish population in the USA (44% of the Jewish population reside in the northeast region [26]), and the GD mutation is more prevalent within the Ashkenazi Jewish population [18]. In contrast, despite the west region being the region with the second highest Jewish population, this was not, in this case, identified as a predictor of GD; the limited number of patients with GD from the west region (61 [8.1%]) in our study may partially account for this observation.

Although the diagnosis of GD could not be confirmed beyond the EHR in the real-world dataset, the characteristics of patients with diagnosed GD in Optum’s de-identified Integrated Claims-Clinical dataset appear consistent with those identified in registries, patient-chart reviews, and population-based cohorts [27–30]: patients with GD had a high prevalence (≥ 15% prevalence in our study) of anemias, thrombocytopenia, splenomegaly, hepatomegaly, bone density disorders, and osteoarthritis. Other common general signs identified included abdominal pain, fatigue, fever, and vitamin D deficiency, as well as depression. Therefore, we are confident the algorithms were trained on appropriate patients.

Both the age- and prevalence-based algorithms developed were more efficient in identifying patients with diagnosed GD in the integrated dataset than the clinical diagnostic algorithm (1204 and 2862 patients assessed, respectively, vs. 20,743 to identify 28 with diagnosed GD), supporting their use in identifying likely candidates for confirmatory diagnostic testing. Patients identified with the clinical diagnostic algorithm tended to have more comorbidities compared to the two algorithms developed by machine learning, which were not all related to or hallmarks of GD, but possibly associated with older age. For example, they had a much higher prevalence of osteoarthritis and hearing impairment. However, they had much lower prevalence of splenomegaly, an important GD hallmark. In general, the age- and prevalence-based models appear to identify younger patients, with profiles closer to the entire GD cohort than the clinical diagnostic algorithm. This was clear in the description of the “highly suspected population” with GD, where those identified with the age-based algorithm were younger than the entire GD cohort and those identified with the prevalence-based algorithm had more pronounced disease manifestations.

Joint application of the age- and prevalence-based algorithms maximizes the identification of patients with GD. Although the implementation of the clinical diagnostic algorithm also would help identify more patients with GD, a substantially higher number of patients would need to be assessed, and as such would require extensive resources for additional testing, which would not benefit a large number. In addition, rule-based filtering classification algorithms such as the clinical diagnostic algorithm are of limited use with data containing missing values [31], and real-world medical datasets typically suffer from such missing data [32]. In contrast, machine learning algorithms, such as those developed here, have the ability to recognize relevant characteristic disease patterns from existing individual patients’ histories despite incomplete/missing data.

Our study has some limitations due to the nature of EHR data. Although we included earliest age at diagnosis, it was not possible from the integrated database to precisely determine the first date diagnosis, which could bias the age of diagnosis toward higher values. Other limitations inherent to use of EHR datasets include information and selection bias as a result of missing/variable information including inequities in healthcare access across socioeconomic status and race, and/or changes in data recording overtime. The accuracy of ICD-10 code E75.22 for GD in EHR databases is unknown, although we required at least 2 codes to reduce biases due to coding errors. Our strict case definition, restricted to those diagnosed with GD or on a GD-specific treatment, and exclusion of those with < 1-year coverage, likely reduced the size of the GD cohort compared to the expected general prevalence and may limit generalizability. EHR databases may not fully capture the clinical manifestations and complications of GD over time. For example, some symptoms such as Erlenmeyer flask deformity were not as prevalent as expected, which may be related to difficulties in capturing such signs in an EHR database.

Ideally, an algorithm(s) would be able to identify undiagnosed patients with GD (e.g. among controls in our study since they had no GD diagnosis). We analyzed de-identified data to develop the algorithms, and as such, we were unable to contact those highly suspected of having undiagnosed GD for further diagnostic testing to assess how well the algorithms identify such patients. Therefore, we can only assess the algorithms’ performance based on identification of diagnosed GD patients, and similarity of highly-suspected patients to the diagnosed GD population. Planned future applications of the age- and prevalence-based algorithms in healthcare systems will remove this limitation.

Although the diagnosed GD cohort in the training and test datasets were both representative of the GD cohort overall, since only 100 patients were included in the test dataset, some dissimilarities may emerge due to the small sample size which could impact algorithm performance. In addition, the construction of the algorithm and features during training affects the information favored by the algorithm to separate the distribution of patients with GD and controls. For example, the prevalence-based algorithm, which included patient features as flags (presence/absence), used clinical characteristics such as hepatomegaly, splenomegaly and thrombocytopenia that had a high prevalence difference between the diagnosed GD and control cohorts to make predictions. It also favored those with an accumulation of symptoms, and thus, biased the algorithm towards older patients. To avoid the bias towards older patients with more disease manifestations, we did not match controls on age with the diagnosed GD cohort so as to teach the algorithm the difference between GD-related morbidity and age-related morbidity. The age-based algorithm favored symptoms with different age of onset between the diagnosed GD and control cohorts. Thus, the age-based algorithm favored patients with earlier onset of GD, i.e. younger patients. Nonetheless, both algorithms performed equivalently in terms of AUPRC, and may be considered complementary since they identify patients across the spectrum of heterogeneity of GD. The use of both algorithms would better capture the heterogeneity inherent to GD, but more research would be needed to compare results between the GD phenotypes (Types 1, 2 and 3).

## Conclusions

Both the age- and prevalence-based algorithms developed are more efficient in identifying patients with diagnosed GD than the existing clinical diagnostic algorithm as applied to a US EHR dataset. These algorithms could shorten diagnostic delay by identifying patients who are appropriate candidates for GD diagnostic testing (e.g. patients highly suspected of GD by the algorithms who do not already have a GD diagnosis).

### Supplementary Information

**Additional file 1:** Supplemental Material.

## Data Availability

The datasets used and/or analyzed during the current study were obtained using standard contracts and data use agreements. The de-identified Integrated Claims-Clinical dataset are proprietary to Optum and, therefore, cannot be broadly disclosed or made publicly available at this time. The disclosure of these data to third-parties would require a data use agreement with Optum.

## References

[1] Platt FM, d'Azzo A, Davidson BL, Neufeld EF, Tifft CJ (2018). Lysosomal storage diseases. Nat Rev Dis Primers.

[2] Parenti G, Medina DL, Ballabio A (2021). The rapidly evolving view of lysosomal storage diseases. EMBO Mol Med.

[3] Wenger DA, Coppola S, Liu SL (2003). Insights into the diagnosis and treatment of lysosomal storage diseases. Arch Neurol.

[4] Tanpaiboon P (2019). Practical management of lysosomal storage disorders (LSDs). Transl Sci Rare Dis.

[5] Stirnemann J, Belmatoug N, Camou F, Serratrice C, Froissart R, Caillaud C (2017). A review of gaucher disease pathophysiology, clinical presentation and treatments. Int J Mol Sci.

[6] Messner MC, Cabot MC (2010). Glucosylceramide in humans. Adv Exp Med Biol.

[7] Bohra V, Nair V (2011). Gaucher’s disease. Indian J Endocrinol Metab.

[8] Linari S, Castaman G (2015). Clinical manifestations and management of Gaucher disease. Clin Cases Miner Bone Metab.

[9] Motta I, Consonni D, Stroppiano M, Benedetto C, Cassinerio E, Tappino B (2021). Predicting the probability of Gaucher disease in subjects with splenomegaly and thrombocytopenia. Sci Rep.

[10] National Organization for Rare Disorders (NORD). Gaucher disease. 2018. https://rarediseases.org/rare-diseases/gaucher-disease/. Accessed 4 May 2022.

[11] European Medicines Agency. Gaucher disease. A strategic collaborative approach from EMA and FDA (EMA/44410/2014). 2014. https://www.ema.europa.eu/en/documents/regulatory-procedural-guideline/gaucher-disease-strategic-collaborative-approach-european-medicines-agency-food-drug-administration_en.pdf. Accessd 4 May 2022.

[12] Wang RY, Bodamer OA, Watson MS, Wilcox WR (2011). ACMG Work Group on Diagnostic Confirmation of Lysosomal Storage Diseases. Lysosomal storage diseases: diagnostic confirmation and management of presymptomatic individuals. Genet Med.

[13] Fuller M, Meikle PJ, Hopwood JJ, Mehta A, Beck M, Sunder-Plassmann G (2006). Epidemiology of lysosomal storage diseases: an overview. Fabry disease: perspectives from 5 years of FOS.

[14] Mistry PK, Sadan S, Yang R, Yee J, Yang M (2007). Consequences of diagnostic delays in type 1 Gaucher disease: the need for greater awareness among hematologists-oncologists and an opportunity for early diagnosis and intervention. Am J Hematol.

[15] Mehta A, Belmatoug N, Bembi B, Deegan P, Elstein D, Göker-Alpan Ö (2017). Exploring the patient journey to diagnosis of Gaucher disease from the perspective of 212 patients with Gaucher disease and 16 Gaucher expert physicians. Mol Genet Metab.

[16] Andrade-Campos M, Alfonso P, Irun P, Armstrong J, Calvo C, Dalmau J (2017). Diagnosis features of pediatric Gaucher disease patients in the era of enzymatic therapy, a national-base study from the Spanish Registry of Gaucher Disease. Orphanet J Rare Dis.

[17] Grosse SD, Rogowski WH, Ross LF, Cornel MC, Dondorp WJ, Khoury MJ (2010). Population screening for genetic disorders in the 21st century: evidence, economics, and ethics. Public Health Genomics.

[18] Mistry PK, Cappellini MD, Lukina E, Ozsan H, Mach Pascual S, Rosenbaum H (2011). A reappraisal of Gaucher disease-diagnosis and disease management algorithms. Am J Hematol.

[19] Genetic and Rare Diseases Information Center (GARD). Gaucher disease. 2021. https://rarediseases.info.nih.gov/diseases/8233/gaucher-disease#:~:text=Gaucher. Accessed 4 Feb 2022. 10.1080/02763869.2022.2131143 36394913

[20] Nielsen F, Nielsen F (2016). Hierarchical Clustering. Introduction to HPC with MPI for data science.

[21] Müllner D. Modern hierarchical, agglomerative clustering algorithms. arXiv:1109.2378. 2011. https://arxiv.org/abs/1109.2378.

[22] Ke G, Meng Q, Finley T, Wang T, Chen W, Ma W, Guyon I, Luxburg UV, Bengio SS, Wallach H, Fergus R, Vishwanathan S (2017). LightGBM: a highly efficient gradient boosting decision tree. Advances in neural information processing systems.

[23] Qi Q, Luo Y, Xu Z, Ji S, Yang T. Stochastic optimization of areas under precision-recall curves with provable convergence. In: 35th conference on neural information processing systems (NeurIPS 2021). 2021. https://proceedings.neurips.cc/paper/2021/file/0dd1bc593a91620daecf7723d2235624-Paper.pdf. Accessed 31 Mar 2022.

[24] Lundberg SM, Lee S-I. A unified approach to interpreting model predictions. In: 31st conference on neural information processing systems (NIPS 2017), Long Beach, CA, USA. 2017. https://arxiv.org/pdf/1705.07874.pdf. Accessed 27 Sept 2021.

[25] Saito T, Rehmsmeier M (2015). The precision-recall plot is more informative than the ROC plot when evaluating binary classifiers on imbalanced datasets. PLoS ONE.

[26] Dashefsky A, Sheskin IM, Dashefsky A, Sheskin IM (2016). Jewish population in the United States, 2015. American Jewish Year Book 2015. The annual record of the North American Jewish Communities.

[27] Jaffe DH, Flaks-Manov N, Benis A, Gabay H, DiBonaventura M, Rosenbaum H (2019). Population-based cohort of 500 patients with Gaucher disease in Israel. BMJ Open.

[28] Charrow J, Andersson HC, Kaplan P, Kolodny EH, Mistry P, Pastores G (2000). The Gaucher registry: demographics and disease characteristics of 1698 patients with Gaucher disease. Arch Intern Med.

[29] Stirnemann J, Vigan M, Hamroun D, Heraoui D, Rossi-Semerano L, Berger MG (2012). The French Gaucher's disease registry: clinical characteristics, complications and treatment of 562 patients. Orphanet J Rare Dis.

[30] Yu CY, Wasim S, Amato D (2018). Gaucher disease in Ontario, Canada: clinical manifestations, natural progression, and treatment response. J Rare Dis Res Treat.

[31] Tran CT, Zhang M, Andreae P, Xue B, Bui LT. An ensemble of rule-based classifiers for incomplete data. In: 21st Asia Pacific symposium on intelligent and evolutionary systems (IES), 2017, pp. 7–12.

[32] Wang H, Tang J, Wu M, Wang X, Zhang T (2022). Application of machine learning missing data imputation techniques in clinical decision making: taking the discharge assessment of patients with spontaneous supratentorial intracerebral hemorrhage as an example. BMC Med Inform Decis Mak.

