# Supplementary material for: Development of a rare disease algorithm to identify persons at risk of Gaucher disease using electronic health records in the United States
Source: Orphanet J Rare Dis. 2023 Sep 9;18:280. doi: 10.1186/s13023-023-02868-2 (PMC10492341; doi:10.1186/s13023-023-02868-2)

**SUPPLEMENTARY MATERIALS**

**Table S1.** GD treatments codes used: Healthcare Common Procedure Coding System [HCPCS] and National Drug Code [NDC].

| **Treatment** | **Healthcare Common Procedure Coding System [HCPCS]** | **National Drug Code [NDC]** |
| --- | --- | --- |
| Cerezyme/Imiglucerase | J1785 or J1786 | 58468198301, 58468466301 or 54868198301 |
| Eliglustat/Cerdelga |  | 58468022002 or 58468022001 |
| Taliglucerase alfa/Elelyso | C9294 or J3060 | 00069010601 |
| Velaglucerase alfa/ VPRIV | J3385 or C9271 | 54092070102 or 54092070104 |

**Table S2.** Demographics of the “highly suspected population” (threshold >0.95) with GD identified by the age- and prevalence-based algorithms compared with those identified using the clinical diagnostic algorithm as having suspected GD, and those diagnosed with GD.

|  | **Age-based**  **N=1073** | **Prevalence-based**  **N=1793** | **Clinical diagnostic algorithm N=20743** | **Entire diagnosed GD cohort N=815** |
| --- | --- | --- | --- | --- |
| **Age at index (years)** |  |  |  |  |
| Mean (SD) | 36 (20) | 52 (21) | 61 (19) | 44 (22) |
| Min | 0 | 0 | 0 | 0 |
| Median (Q1-Q3) | 37 (20-49) | 56 (38-69) | 64 (51-76) | 45 (27-60) |
| Max | 84 | 88 | 88 | 87 |
| **Age in class** |  |  |  |  |
| 0–9 | 109 (10.2%) | 47 (2.6%) | 282 (1.4%) | 62 (7.6%) |
| 10–19 | 148 (13.8%) | 97 (5.4%) | 442 (2.1%) | 62 (7.6%) |
| 20–29 | 145 (13.5%) | 155 (8.6%) | 1175 (5.7%) | 105 (12.9%) |
| 30–39 | 165 (15.4%) | 176 (9.8%) | 1333 (6.4%) | 117 (14.4%) |
| 40–49 | 258 (24.0%) | 241 (13.4%) | 1673 (8.1%) | 119 (14.6%) |
| 50–59 | 94 (8.8%) | 315 (17.6%) | 3436 (16.6%) | 130 (16.0%) |
| 60–69 | 79 (7.4%) | 339 (18.9%) | 4279 (20.6%) | 118 (14.5%) |
| 70–79 | 70 (6.5%) | 270 (15.1%) | 4688 (22.6%) | 76 (9.3%) |
| 80–89 | 5 (0.5%) | 153 (8.5%) | 3435 (16.6%) | 26 (3.2%) |
| **Race** |  |  |  |  |
| African American | 63 (5.9%) | 68 (3.8%) | 2623 (12.6%) | 30 (3.7%) |
| Asian | 6 (0.6%) | 4 (0.2%) | 349 (1.7%) | 2 (0.2%) |
| Caucasian | 850 (79.2%) | 1507 (84.0%) | 16316 (78.7%) | 672 (82.5%) |
| Other/Unknown | 154 (14.4%) | 214 (11.9%) | 1455 (7.0%) | 111 (13.6%) |
| **Ethnicity** |  |  |  |  |
| Hispanic | 101 (9.4%) | 110 (6.1%) | 1133 (5.5%) | 38 (4.7%) |
| Not Hispanic | 838 (78.1%) | 1480 (82.5%) | 18183 (87.7%) | 664 (81.5%) |
| Unknown | 134 (12.5%) | 203 (11.3%) | 1427 (6.9%) | 113 (13.9%) |
| **Region** |  |  |  |  |
| Midwest | 304 (28.3%) | 451 (25.2%) | 10004 (48.2%) | 198 (24.3%) |
| Northeast | 374 (34.9%) | 786 (43.8%) | 2334 (11.3%) | 373 (45.8%) |
| South | 259 (24.1%) | 316 (17.6%) | 5970 (28.8%) | 153 (18.8%) |
| West | 91 (8.5%) | 183 (10.2%) | 1696 (8.2%) | 66 (8.1%) |
| Other/Unknown | 45 (4.2%) | 57 (3.2%) | 739 (3.6%) | 25 (3.1%) |
| **Death** |  |  |  |  |
| 0 | 976 (91.0%) | 1553 (86.6%) | 14589 (70.3%) | 772 (94.7%) |
| 1 | 97 (9.0%) | 240 (13.4%) | 6154 (29.7%) | 43 (5.3%) |
| **Coverage (years)** |  |  |  |  |
| Mean (SD) | 7 (4) | 8 (3) | 8 (4) | 6 (4) |
| Min | 0 | 0 | 0 | 0 |
| Median (Q1-Q3) | 7 (3-10) | 9 (5-11) | 8 (5-11) | 7 (3-9) |
| Max | 12.75 | 12.75 | 12.75 | 12.75 |
| **Look-back period (years)** |  |  |  |  |
| Mean (SD) | 3 (2) | 4 (2) | 3 (2) | 3 (3) |
| Min | 0 | 0 | 0 | 0 |
| Median (Q1-Q3) | 2 (1-5) | 3 (2-6) | 3 (2-6) | 2 (0-5) |
| Max | 11.47 | 11.47 | 11.47 | 12.36 |
| **Look-back period** |  |  |  |  |
| at least 6 months | 815 (76.0%) | 1627 (90.7%) | 17593 (84.8%) | 574 (70.4%) |
| at least 12 months | 747 (69.6%) | 1495 (83.4%) | 16552 (79.8%) | 513 (62.9%) |
| at least 18 months | 743 (69.2%) | 1493 (83.3%) | 16549 (79.8%) | 452 (55.5%) |
| at least 24 months | 594 (55.4%) | 1353 (75.5%) | 13683 (66.0%) | 404 (49.6%) |
| at least 36 months | 333 (31.0%) | 781 (43.6%) | 8621 (41.6%) | 325 (39.9%) |
| **Number of distinct symptoms** |  |  |  |  |
| Mean (SD) | 6 (4) | 5 (3) | 7 (3) | 5 (4) |
| Min | 0 | 0 | 0 | 0 |
| Median (Q1-Q3) | 5 (3-8) | 5 (3-7) | 6 (4-9) | 4 (2-7) |
| Max | 24 | 24 | 24 | 19 |
| **Rare disease history** |  |  |  |  |
| 0 | 1073 (100.0%) | 1793 (100.0%) | 20742 (100.0%) | 815 (100.0%) |
| 1 | 0 (0%) | 0 (0%) | 1 (0%) | 0 (0%) |

**Table S3.** Clinical characteristics of the “highly suspected population” (threshold >0.95) identified by the age- and prevalence-based algorithms compared with those identified using the clinical diagnostic algorithm as having suspected GD, and those diagnosed with GD.

| **Features** | **Age-based**  **N=1073** | | | **Prevalence-based**  **N=1793** | | | **Clinical diagnostic algorithm**  **N=20743** | | | **Entire diagnosed GD cohort N=815** | | |
| --- | --- | --- | --- | --- | --- | --- | --- | --- | --- | --- | --- | --- |
|  | **N (%)** | **Age at 1st event; years, mean (SD)** | | **N (%)** | **Age at 1st event; years, mean (SD)** | | **N (%)** | | **Age at 1st event; years, mean (SD)** | **N (%)** | | **Age at 1st event; years, mean (SD)** |
| **Organomegaly** |  | |  | | |  | |  | | |  | |
| Hepatomegaly | 196 (18.3%) | 29 (19) | | 288 (16.1%) | 47 (22) | | 880 (4.2%) | | 57 (18) | 136 (16.7%) | | 35 (22) |
| Splenomegaly | 589 (54.9%) | 35 (18) | | 762 (42.5%) | 48 (21) | | 1526 (7.4%) | | 56 (19) | 278 (34.1%) | | 39 (21) |
| Ventriculomegaly | 0 (0%) |  | | 1 (0.1%) |  | | 4 (0%) | | 57 (38) | 2 (0.2%) | | 74 (0) |
| **Anemia** |  | |  | | |  | |  | | |  | |
| Anemias | 903 (84.2%) | 37 (19) | | 1616 (90.1%) | 53 (20) | | 20245 (97.6%) | | 60 (19) | 642 (78.8%) | | 43 (21) |
| **Blood disorder** |  | |  | | |  | |  | | |  | |
| Coagulation defects | 84 (7.8%) | 51 (22) | | 89 (5.0%) | 55 (19) | | 1139 (5.5%) | | 62 (18) | 31 (3.8%) | | 49 (25) |
| Pancytopenia | 102 (9.5%) | 37 (20) | | 120 (6.7%) | 55 (21) | | 1433 (6.9%) | | 63 (17) | 33 (4.0%) | | 43 (25) |
| Thrombocytopenia | 600 (55.9%) | 39 (19) | | 940 (52.4%) | 56 (20) | | 18162 (87.6%) | | 62 (19) | 364 (44.7%) | | 43 (22) |
| **Bone disorder** |  | |  | | |  | |  | | |  | |
| Arthralgia | 36 (3.4%) | 43 (16) | | 61 (3.4%) | 56 (16) | | 948 (4.6%) | | 63 (16) | 19 (2.3%) | | 48 (18) |
| Arthrogryposis | 19 (1.8%) | 43 (24) | | 32 (1.8%) | 57 (20) | | 614 (3.0%) | | 63 (16) | 9 (1.1%) | | 68 (11) |
| Avascular necrosis | 40 (3.7%) | 37 (18) | | 30 (1.7%) | 49 (20) | | 130 (0.6%) | | 62 (17) | 31 (3.8%) | | 48 (18) |
| Bone density disorders | 204 (19.0%) | 54 (16) | | 273 (15.2%) | 65 (15) | | 3924 (18.9%) | | 71 (12) | 234 (28.7%) | | 55 (17) |
| Bone pain | 72 (6.7%) | 42 (17) | | 101 (5.6%) | 53 (19) | | 1978 (9.5%) | | 50 (21) | 68 (8.3%) | | 44 (17) |
| Chondropathies | 14 (1.3%) | 52 (13) | | 27 (1.5%) | 55 (22) | | 331 (1.6%) | | 60 (17) | 10 (1.2%) | | 52 (24) |
| Delayed skeletal maturation | 19 (1.8%) | 45 (23) | | 13 (0.7%) | 57 (15) | | 322 (1.6%) | | 65 (16) | 11 (1.3%) | | 48 (22) |
| Erlenmeyer flask deformity | 3 (0.3%) | 41 (21) | | 3 (0.2%) | 34 (17) | | 39 (0.2%) | | 61 (15) | 0 (0%) | |  |
| Joint dislocation | 8 (0.7%) | 42 (28) | | 15 (0.8%) | 60 (25) | | 122 (0.6%) | | 62 (17) | 2 (0.2%) | | 58 (5) |
| Kyphosis | 52 (4.8%) | 48 (22) | | 43 (2.4%) | 63 (19) | | 457 (2.2%) | | 72 (15) | 15 (1.8%) | | 43 (29) |
| Osteoarthritis | 184 (17.1%) | 57 (17) | | 423 (23.6%) | 64 (14) | | 7558 (36.4%) | | 69 (12) | 152 (18.7%) | | 60 (15) |
| Osteolysis | 1 (0.1%) |  | | 1 (0.1%) |  | | 39 (0.2%) | | 70 (15) | 5 (0.6%) | | 66 (7) |
| Osteonecrosis | 73 (6.8%) | 38 (16) | | 80 (4.5%) | 55 (19) | | 180 (0.9%) | | 60 (17) | 53 (6.5%) | | 47 (18) |
| Osteopenia | 64 (6.0%) | 45 (17) | | 69 (3.8%) | 64 (15) | | 959 (4.6%) | | 70 (13) | 78 (9.6%) | | 48 (19) |
| Osteoporosis | 61 (5.7%) | 54 (18) | | 76 (4.2%) | 67 (15) | | 1410 (6.8%) | | 72 (12) | 61 (7.5%) | | 57 (17) |
| Pathological fracture | 20 (1.9%) | 55 (18) | | 24 (1.3%) | 62 (19) | | 421 (2.0%) | | 70 (16) | 11 (1.3%) | | 53 (18) |
| Spine deformation | 4 (0.4%) | 32 (33) | | 6 (0.3%) | 56 (24) | | 48 (0.2%) | | 61 (22) | 0 (0%) | |  |
| **Cerebral/nervous system disorder** |  | |  | | |  | |  | | |  | |
| Ataxia | 8 (0.7%) | 48 (34) | | 44 (2.5%) | 63 (19) | | 562 (2.7%) | | 68 (15) | 11 (1.3%) | | 53 (28) |
| Bradykinesia | 4 (0.4%) | 71 (1) | | 9 (0.5%) | 74 (10) | | 35 (0.2%) | | 74 (10) | 4 (0.5%) | | 62 (11) |
| Cranial nerve disorders | 1 (0.1%) |  | | 0 (0%) |  | | 32 (0.2%) | | 61 (19) | 1 (0.1%) | |  |
| Developmental regression | 101 (9.4%) | 35 (24) | | 134 (7.5%) | 54 (25) | | 3603 (17.4%) | | 65 (20) | 52 (6.4%) | | 36 (28) |
| Dysphagia | 77 (7.2%) | 52 (24) | | 184 (10.3%) | 59 (20) | | 3416 (16.5%) | | 68 (16) | 57 (7.0%) | | 50 (27) |
| Extrapyramidal disorder | 4 (0.4%) | 52 (23) | | 10 (0.6%) | 58 (21) | | 138 (0.7%) | | 61 (18) | 5 (0.6%) | | 53 (15) |
| Gaze Palsy | 4 (0.4%) | 51 (24) | | 1 (0.1%) |  | | 16 (0.1%) | | 56 (27) | 1 (0.1%) | |  |
| Hearing impairment | 108 (10.1%) | 46 (26) | | 189 (10.5%) | 64 (19) | | 3547 (17.1%) | | 70 (16) | 67 (8.2%) | | 55 (23) |
| Hemiplegia/Hemiparesis | 14 (1.3%) | 55 (23) | | 22 (1.2%) | 64 (18) | | 760 (3.7%) | | 67 (17) | 7 (0.9%) | | 55 (22) |
| Hydrocephalus | 3 (0.3%) | 12 (9) | | 19 (1.1%) | 66 (21) | | 219 (1.1%) | | 59 (25) | 6 (0.7%) | | 60 (29) |
| Laryngeal spasm | 4 (0.4%) | 36 (39) | | 0 (0%) |  | | 25 (0.1%) | | 65 (20) | 3 (0.4%) | | 29 (41) |
| Muscle hypotonia | 15 (1.4%) | 7 (6) | | 6 (0.3%) | 4 (3) | | 40 (0.2%) | | 14 (25) | 7 (0.9%) | | 3 (2) |
| Myoclonic seizure | 3 (0.3%) | 24 (17) | | 5 (0.3%) | 24 (12) | | 11 (0.1%) | | 46 (22) | 1 (0.1%) | |  |
| Nerve root compression | 0 (0%) |  | | 1 (0.1%) |  | | 29 (0.1%) | | 64 (16) | 0 (0%) | |  |
| Oculomotor apraxia | 1 (0.1%) |  | | 1 (0.1%) |  | | 14 (0.1%) | | 56 (23) | 4 (0.5%) | | 28 (36) |
| Opticokinetic nystagmus | 5 (0.5%) | 5 (6) | | 8 (0.4%) | 26 (25) | | 89 (0.4%) | | 57 (24) | 5 (0.6%) | | 26 (32) |
| Paralytic strabismus | 1 (0.1%) |  | | 4 (0.2%) | 47 (23) | | 32 (0.2%) | | 39 (31) | 1 (0.1%) | |  |
| Parkinson | 47 (4.4%) | 65 (11) | | 99 (5.5%) | 74 (9) | | 340 (1.6%) | | 74 (10) | 23 (2.8%) | | 65 (11) |
| Tonic clonic seizure | 3 (0.3%) | 33 (11) | | 8 (0.4%) | 39 (23) | | 47 (0.2%) | | 53 (20) | 2 (0.2%) | | 12 (4) |
| **Development disorders** |  | |  | | |  | |  | | |  | |
| Delayed puberty | 4 (0.4%) | 17 (6) | | 2 (0.1%) | 15 (1) | | 5 (0%) | | 25 (24) | 1 (0.1%) | |  |
| Growth retardation | 0 (0%) |  | | 1 (0.1%) |  | | 20 (0.1%) | | 8 (11) | 6 (0.7%) | | 15 (13) |
| Short stature | 10 (0.9%) | 11 (4) | | 7 (0.4%) | 12 (4) | | 50 (0.2%) | | 16 (19) | 7 (0.9%) | | 11 (4) |
| **Eye disorder** |  | |  | | |  | |  | | |  | |
| Corneal disorders | 12 (1.1%) | 57 (27) | | 15 (0.8%) | 60 (22) | | 130 (0.6%) | | 70 (17) | 7 (0.9%) | | 49 (32) |
| Non-diabetic retinopathy | 5 (0.5%) | 41 (30) | | 12 (0.7%) | 53 (23) | | 190 (0.9%) | | 58 (26) | 3 (0.4%) | | 61 (26) |
| **General signs** |  | |  | | |  | |  | | |  | |
| Abdominal pain | 398 (37.1%) | 40 (20) | | 637 (35.5%) | 50 (21) | | 10775 (51.9%) | | 60 (19) | 218 (26.7%) | | 47 (22) |
| Elevated CRP | 10 (0.9%) | 47 (24) | | 9 (0.5%) | 54 (20) | | 197 (0.9%) | | 59 (20) | 6 (0.7%) | | 52 (15) |
| Fatigue | 376 (35.0%) | 41 (18) | | 571 (31.8%) | 54 (19) | | 8895 (42.9%) | | 63 (17) | 227 (27.9%) | | 47 (19) |
| Fever | 309 (28.8%) | 34 (20) | | 399 (22.3%) | 48 (22) | | 7879 (38.0%) | | 59 (20) | 147 (18.0%) | | 40 (23) |
| Gingival bleeding | 2 (0.2%) | 34 (17) | | 6 (0.3%) | 58 (20) | | 29 (0.1%) | | 52 (25) | 2 (0.2%) | | 51 (32) |
| Muscle atrophy | 1 (0.1%) |  | | 0 (0%) |  | | 11 (0.1%) | | 74 (8) | 4 (0.5%) | | 77 (12) |
| Tremor | 55 (5.1%) | 51 (21) | | 88 (4.9%) | 59 (19) | | 1438 (6.9%) | | 64 (17) | 31 (3.8%) | | 51 (24) |
| Vitamin D deficiency | 177 (16.5%) | 48 (20) | | 246 (13.7%) | 57 (18) | | 3205 (15.5%) | | 64 (17) | 135 (16.6%) | | 51 (18) |
| **Heart disorder** |  | |  | | |  | |  | | |  | |
| Valve calcification | 12 (1.1%) | 65 (13) | | 18 (1.0%) | 68 (14) | | 772 (3.7%) | | 72 (13) | 13 (1.6%) | | 66 (20) |
| **Hepatic disorders** |  | |  | | |  | |  | | |  | |
| Cirrhosis | 52 (4.8%) | 43 (13) | | 74 (4.1%) | 56 (15) | | 1324 (6.4%) | | 62 (13) | 14 (1.7%) | | 46 (16) |
| Hepatic fibrosis | 4 (0.4%) | 19 (18) | | 11 (0.6%) | 50 (24) | | 61 (0.3%) | | 61 (14) | 6 (0.7%) | | 48 (29) |
| Hepatitis | 40 (3.7%) | 39 (15) | | 50 (2.8%) | 52 (18) | | 641 (3.1%) | | 60 (16) | 19 (2.3%) | | 49 (23) |
| Portal hypertension | 50 (4.7%) | 37 (14) | | 42 (2.3%) | 52 (14) | | 667 (3.2%) | | 60 (12) | 13 (1.6%) | | 38 (22) |
| **Immunology** |  | |  | | |  | |  | | |  | |
| Polyclonal gammopathy | 0 (0%) |  | | 4 (0.2%) | 63 (9) | | 53 (0.3%) | | 67 (12) | 4 (0.5%) | | 60 (19) |
| **Kidney disorder** |  | |  | | |  | |  | | |  | |
| Acute kidney disease | 34 (3.2%) | 63 (19) | | 54 (3.0%) | 64 (16) | | 225 (1.1%) | | 63 (19) | 31 (3.8%) | | 66 (14) |
| Hematuria | 130 (12.1%) | 47 (21) | | 260 (14.5%) | 57 (19) | | 4120 (19.9%) | | 65 (17) | 75 (9.2%) | | 53 (20) |
| Proteinuria | 47 (4.4%) | 47 (22) | | 115 (6.4%) | 59 (17) | | 2446 (11.8%) | | 63 (17) | 35 (4.3%) | | 53 (22) |
| **Malignancy** |  | |  | | |  | |  | | |  | |
| Liver neoplasm | 7 (0.7%) | 44 (20) | | 24 (1.3%) | 63 (17) | | 275 (1.3%) | | 66 (12) | 3 (0.4%) | | 55 (26) |
| Malignant melanoma | 4 (0.4%) | 52 (25) | | 7 (0.4%) | 64 (13) | | 188 (0.9%) | | 70 (13) | 5 (0.6%) | | 63 (7) |
| Multiple myeloma | 14 (1.3%) | 51 (18) | | 24 (1.3%) | 67 (14) | | 370 (1.8%) | | 69 (12) | 8 (1.0%) | | 61 (9) |
| Non Hodgkin Lymphoma | 26 (2.4%) | 51 (20) | | 29 (1.6%) | 67 (17) | | 60 (0.3%) | | 69 (12) | 8 (1.0%) | | 61 (27) |
| Other malignant neoplasms | 3 (0.3%) | 56 (35) | | 2 (0.1%) | 43 (38) | | 20 (0.1%) | | 66 (14) | 2 (0.2%) | | 38 (46) |
| Pancreatic cancer | 5 (0.5%) | 43 (22) | | 11 (0.6%) | 70 (13) | | 278 (1.3%) | | 69 (11) | 2 (0.2%) | | 55 (15) |
| Uncertain neoplasms | 68 (6.3%) | 50 (17) | | 131 (7.3%) | 63 (14) | | 1113 (5.4%) | | 67 (16) | 47 (5.8%) | | 58 (16) |
| **Perinatal disorders** |  | |  | | |  | |  | | |  | |
| Hydrops fetalis | 2 (0.2%) | 1 (1) | | 1 (0.1%) |  | | 2 (0%) | | 1 (1) | 1 (0.1%) | |  |
| Ichthyosis | 1 (0.1%) |  | | 1 (0.1%) |  | | 15 (0.1%) | | 71 (10) | 0 (0%) | |  |
| **Psychiatric disorders** |  | |  | | |  | |  | | |  | |
| Dementia non-senile | 33 (3.1%) | 66 (16) | | 114 (6.4%) | 76 (12) | | 2368 (11.4%) | | 78 (9) | 19 (2.3%) | | 69 (16) |
| Depression | 293 (27.3%) | 43 (19) | | 620 (34.6%) | 52 (19) | | 8641 (41.7%) | | 61 (18) | 161 (19.8%) | | 51 (20) |
| **Respiratory disorder** |  | |  | | |  | |  | | |  | |
| Interstitial pulmonary abnormality | 12 (1.1%) | 66 (23) | | 12 (0.7%) | 60 (22) | | 417 (2.0%) | | 69 (14) | 7 (0.9%) | | 44 (36) |
| Pulmonary fibrosis | 14 (1.3%) | 68 (15) | | 41 (2.3%) | 58 (16) | | 953 (4.6%) | | 70 (13) | 13 (1.6%) | | 63 (20) |
| Pulmonary hypertension | 51 (4.8%) | 54 (23) | | 91 (5.1%) | 64 (15) | | 2132 (10.3%) | | 71 (15) | 35 (4.3%) | | 56 (21) |
| Respiratory failure | 66 (6.2%) | 48 (27) | | 103 (5.7%) | 61 (19) | | 5165 (24.9%) | | 67 (16) | 36 (4.4%) | | 56 (30) |

**Table S4.** Visits to the specialist by the “highly suspected population” (threshold >0.95) identified by the age-based and prevalence algorithms compared with those identified using the clinical diagnostic algorithm as having suspected GD, and those diagnosed with GD.

| **Features** | **Age-based**  **N=1073** | | | **Prevalence-based**  **N=1793** | | |
| --- | --- | --- | --- | --- | --- | --- |
|  | **N (%)** | **Age at 1st event; years, mean (SD)** | **No. of visits (mean)** | **N (%)** | **Age at 1st event; years, mean (SD)** | **No. of visits (mean)** |
| Gastroenterology | 288 (26.8%) | 44 (19) | 0.61 | 578 (32.2%) | 56 (18) | 0.37 |
| General Practice | 53 (4.9%) | 45 (19) | 0.03 | 207 (11.5%) | 53 (20) | 0.05 |
| Hematology | 40 (3.7%) | 44 (15) | 0.23 | 52 (2.9%) | 53 (18) | 0.05 |
| Hepatology | 21 (2.0%) | 41 (9) | 0.04 | 29 (1.6%) | 53 (12) | 0.02 |
| Internal Medicine | 517 (48.2%) | 45 (17) | 2.11 | 1276 (71.2%) | 55 (19) | 0.84 |
| Neurology | 448 (41.8%) | 41 (21) | 1.89 | 838 (46.7%) | 55 (20) | 0.76 |
| Oncology | 506 (47.2%) | 37 (22) | 2.19 | 754 (42.1%) | 54 (21) | 1.08 |
| Ophthalmology | 157 (14.6%) | 46 (26) | 0.23 | 297 (16.6%) | 56 (21) | 0.07 |
| Orthopedic Surgery | 332 (30.9%) | 42 (23) | 0.53 | 654 (36.5%) | 54 (20) | 0.28 |
| Pain Medicine | 47 (4.4%) | 47 (19) | 0.15 | 117 (6.5%) | 57 (17) | 0.04 |
| Pediatrics | 226 (21.1%) | 15 (15) | 0.83 | 225 (12.5%) | 29 (23) | 0.19 |
| Radiology | 335 (31.2%) | 43 (22) | 0.46 | 783 (43.7%) | 54 (20) | 0.23 |
| Rheumatology | 69 (6.4%) | 46 (20) | 0.1 | 111 (6.2%) | 57 (17) | 0.05 |

**Table S4.** continued.

| **Features** | **Clinical diagnostic algorithm** | | | **Entire diagnosed GD cohort** | | |  |
| --- | --- | --- | --- | --- | --- | --- | --- |
|  | **N=20743** | | | **N=815** | | |  |
|  | **N (%)** | **Age at 1st event; years, mean (SD)** | **No. of visits (with 0)** | **N (%)** | **Age at 1st event; years, mean (SD)** | **No. of visits (with 0)** | |
| Gastroenterology | 8314 (40.1%) | 63 (16) | 0.49 | 194 (23.8%) | 49 (21) | 3.06 | |
| General Practice | 1943 (9.4%) | 62 (18) | 0.12 | 36 (4.4%) | 51 (19) | 0.58 | |
| Hematology | 670 (3.2%) | 63 (16) | 0.05 | 35 (4.3%) | 53 (20) | 0.79 | |
| Hepatology | 300 (1.4%) | 58 (14) | 0.02 | 10 (1.2%) | 53 (16) | 0.02 | |
| Internal Medicine | 16215 (78.2%) | 63 (17) | 3.44 | 420 (51.5%) | 50 (18) | 23.38 | |
| Neurology | 7073 (34.1%) | 62 (18) | 0.56 | 399 (49.0%) | 43 (21) | 49.06 | |
| Oncology | 6777 (32.7%) | 63 (17) | 1.4 | 287 (35.2%) | 45 (22) | 61.71 | |
| Ophthalmology | 4354 (21.0%) | 64 (18) | 0.16 | 128 (15.7%) | 53 (22) | 3.17 | |
| Orthopedic Surgery | 6717 (32.4%) | 64 (17) | 0.39 | 223 (27.4%) | 51 (21) | 5.2 | |
| Pain Medicine | 1124 (5.4%) | 64 (16) | 0.03 | 31 (3.8%) | 54 (17) | 0.19 | |
| Pediatrics | 2106 (10.2%) | 47 (27) | 0.2 | 142 (17.4%) | 20 (21) | 18.14 | |
| Radiology | 9788 (47.2%) | 62 (17) | 0.74 | 248 (30.4%) | 46 (22) | 3.6 | |
| Rheumatology | 2125 (10.2%) | 63 (17) | 0.13 | 99 (12.1%) | 51 (20) | 7.21 | |

**Figure S1.** Features identified for GD algorithm assessment. Overall, 80 clinical characteristics of GD were identified and grouped into organ classes.


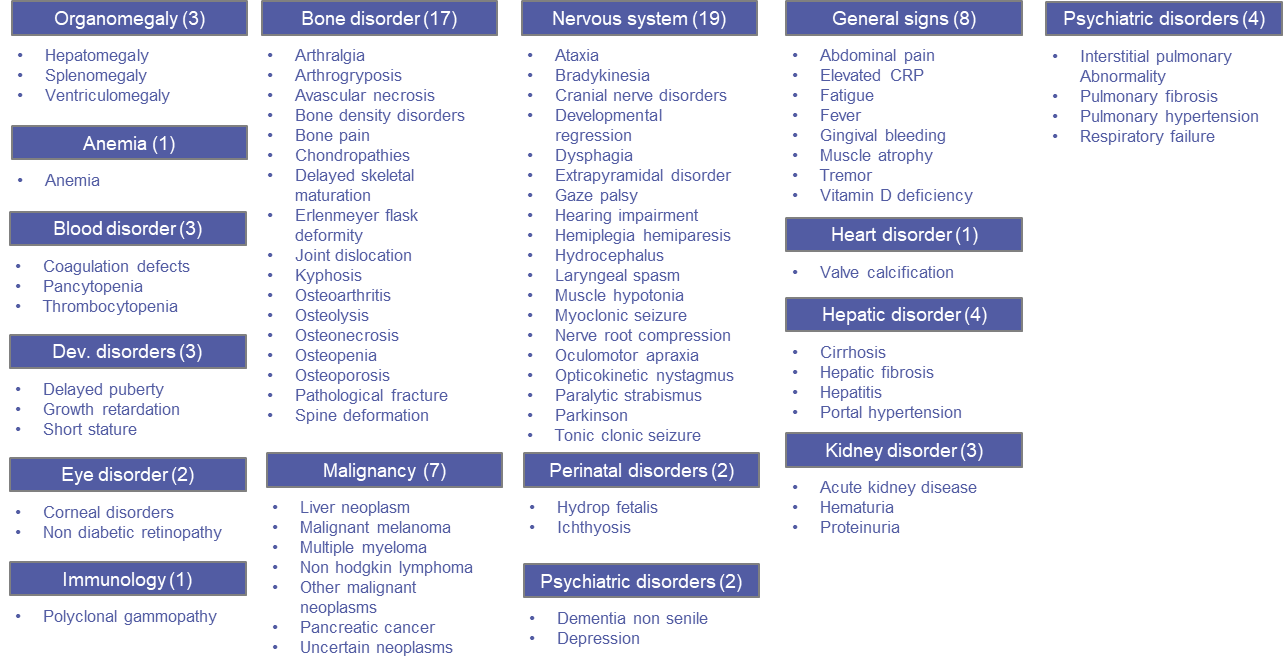


Figure S2. Defining clinical characteristics with symptoms, procedures, laboratory measurement and SDS terms.


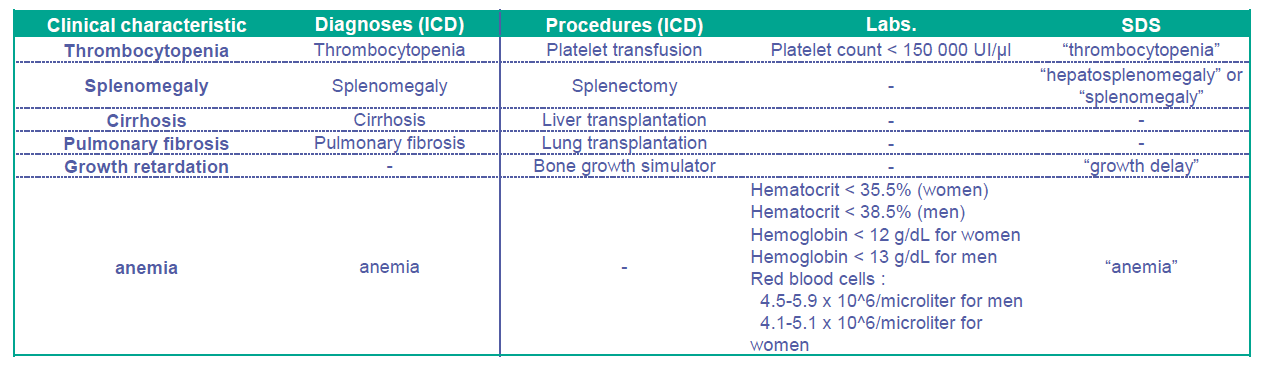


Figure S3. Patients identified using the clinical diagnostic algorithm as having suspected GD.


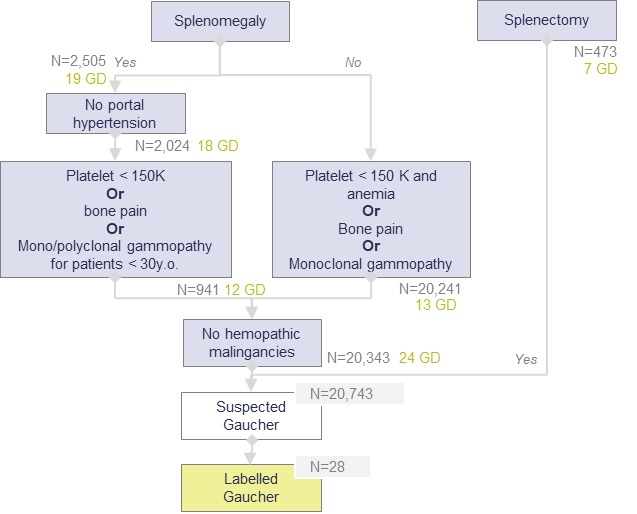


Figure S4. Methodology overview.


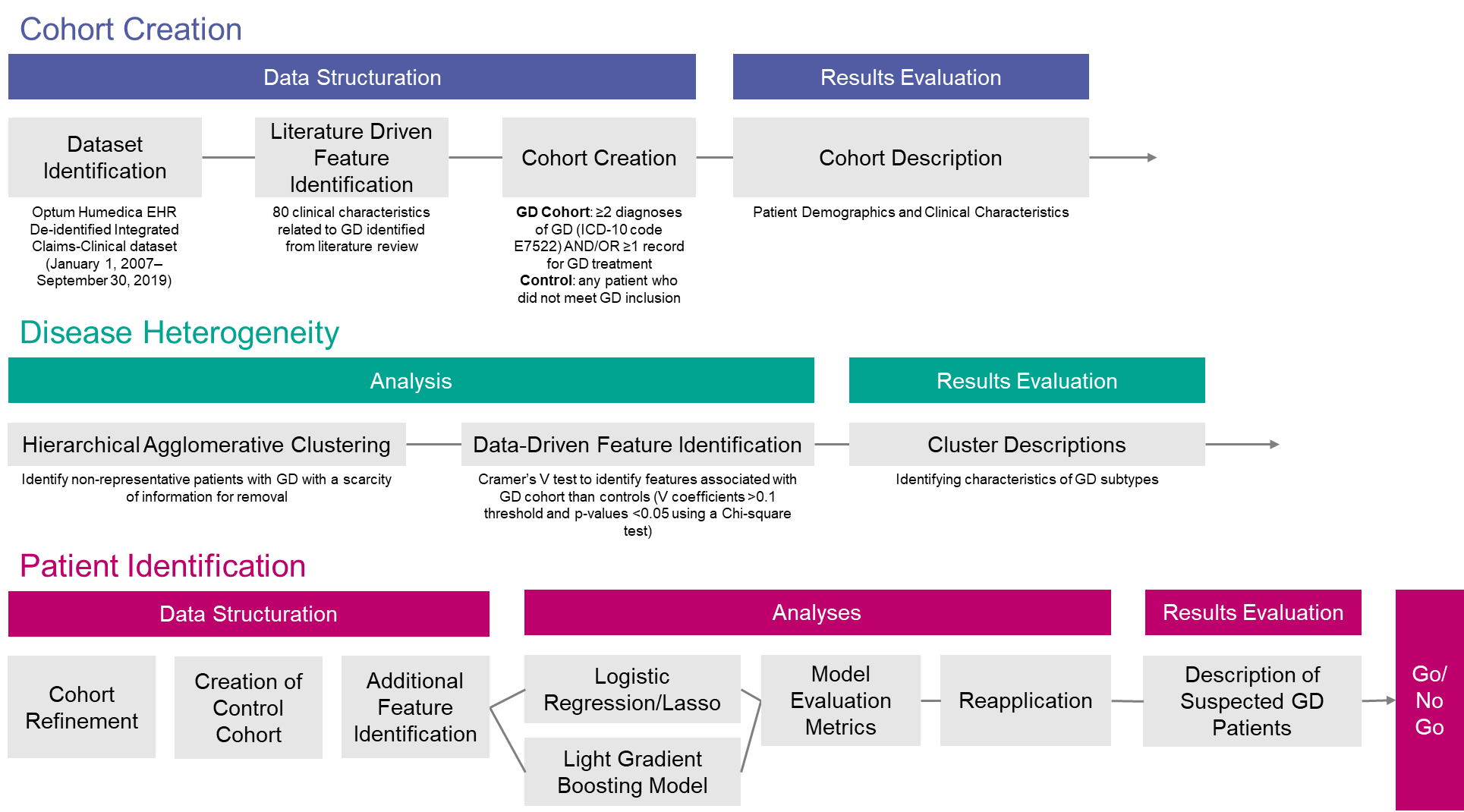

Supplement: Supplementary file 1 — Additional file 1: Supplemental Material. [file 13023_2023_2868_MOESM1_ESM.docx]
